# A 1-nS 1-V Sub-1-µW Linear CMOS OTA with Rail-to-Rail Input for Hz-Band Sensory Interfaces

**DOI:** 10.3390/s20113303

**Published:** 2020-06-10

**Authors:** Jacek Jakusz, Waldemar Jendernalik, Grzegorz Blakiewicz, Miron Kłosowski, Stanisław Szczepański

**Affiliations:** Faculty of Electronics, Gdańsk University of Technology, 80-233 Gdańsk, Poland; jacek.jakusz@pg.edu.pl (J.J.); grzegorz.blakiewicz@pg.edu.pl (G.B.); miron.klosowski@pg.edu.pl (M.K.); stanislaw.szczepanski@pg.edu.pl (S.S.)

**Keywords:** very low frequency, operational transconductance amplifier (OTA), biomedical sensor interface, biomedical electronics, low-voltage low-power electronics, CMOS

## Abstract

The paper presents an operational transconductance amplifier (OTA) with low transconductance (0.62–6.28 nS) and low power consumption (28–270 nW) for the low-frequency analog front-ends in biomedical sensor interfaces. The proposed OTA implements an innovative, highly linear voltage-to-current converter based on the channel-length-modulation effect, which can be rail-to-rail driven. At 1-V supply and 1-V_pp_ asymmetrical input driving, the linearity error in the current-voltage characteristics is 1.5%, while the total harmonic distortion (THD) of the output current is 0.8%. For a symmetrical 2-V_pp_ input drive, the linearity error is 0.3%, whereas THD reaches 0.2%. The linearity is robust for the mismatch and the process-voltage-and-temperature (PVT) variations. The temperature drift of transconductance is 10 pS/°C. The prototype circuit was fabricated in 180-nanometer CMOS technology.

## 1. Introduction

**Figure 1:**
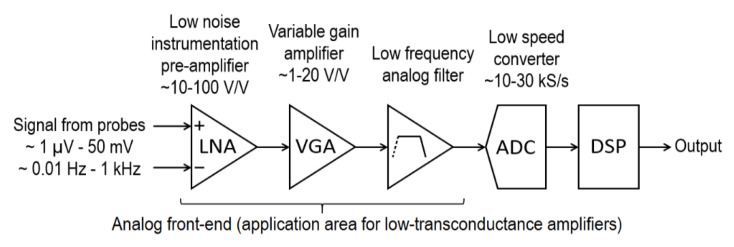
The main components of a microelectronic system processing low-frequency signals.

Operational transconductance amplifiers (OTAs) with a very low conversion ratio of 10^−11^ to 10^−7^ A/V (hereinafter referred to as low-transconductance amplifiers, LTA) are used in analog pre-processing of very-low-frequency biomedical signals. Before digitalization, the analog biomedical signal is amplified and pre-filtered as illustrated in Figure 1 [1,2,3,4,5]. First, the signal is amplified by a low noise amplifier (LNA). Then, the signal amplitude is corrected by a variable gain amplifier (VGA) in order to make it suitable for the input of an analog-to-digital converter (ADC). The analog filter reduces the bandwidth to minimize signal distortion that results from the aliasing effect. LNA, VGA and the filter constitute an analog front-end. It should be noted that the filter does not have to be implemented as an individual block. Instead, the filtering component can be embedded into LNA and VGA through the limitation of their bandwidth [1,6].

The amplitude of biomedical signal ranges from a few µV to several tens of mV, and its frequency is from 0.01 Hz to 3 kHz [6]. To achieve optimal processing dynamics, the front-end is integrated with ADC in a single chip. However, the integration of low-frequency circuits is a challenge. The corner frequency is associated with the RC-time-constant, i.e., *f* ≈ 1/(2*πRC*). In a typical CMOS process, resistors can be made of polysilicon with a resistivity of 10^3^ Ω/square, and capacitors made of polysilicon-insulator-polysilicon or metal-insulator-metal layers with a unit capacitance of 10^−15^ F/µm^2^. Due to large area requirements, the practical values of the resistances and capacitances in integrated circuits are *R* < 100 kΩ and *C* < 100 pF, whereas the corresponding corner frequency is *f* > 15 kHz. For further reduction of frequency, the RC-time-constant can be increased using active techniques such as the capacitance multiplication and resistance emulation. Examples of using the capacitance (Miller’s) multiplication in front-ends can be found in [1,7]. However, the majority of the available literature focuses on LTA-based emulation of large resistances [2,3,8,9,10,11,12,13,14,15,16,17,18]. LTAs emulate resistors as large as 10^7^–10^11^ Ω while occupying a reasonable area of 0.01 mm^2^ to 0.1 mm^2^ [2,3,8,9,10,11,12,13,14,15,16,17,18]. However, when compared to passive components, the performance figures of active resistors such as linearity, temperature stability and noise are worse. Improvement of these parameters is one of the main goals of contemporary research studies [12].

This work presents an innovative LTA that is developed and manufactured in a 0.18-µm CMOS technology at the Taiwan Semiconductor Manufacturing Company (TSMC). LTA uses the so-called Early effect (channel-length-modulation effect) in a field effect transistor (FET) to directly emulate high resistance. The relationship between the output current and the input voltage of LTA is linear from the ground to the supply voltage. The circuit design is relatively simple and does not require special optimization. Owing to the use of sub-microwatt power consumption and 1-V supply, it is suitable for use in low-power low-voltage biomedical interfaces. The following chapters present the operating principle, circuit details, theoretical analysis and prototype measurement results.

## 2. Voltage-to-Current Conversion Using Channel-Length-Modulation Effect

### 2.1. Operation Principles

**Figure 2:**
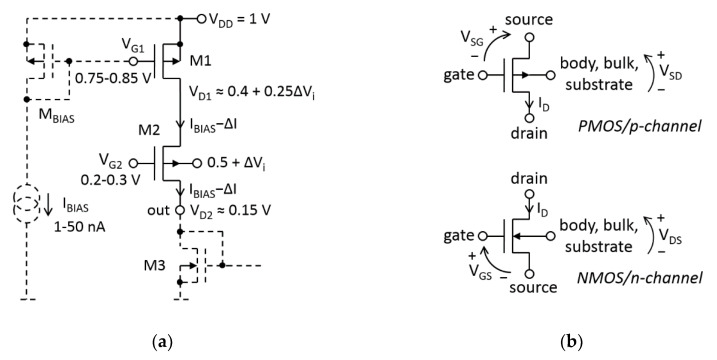
A voltage-to-low-current conversion utilizing the channel-length-modulation effect in MOSFET: (**a**) the realization in CMOS technology; (**b**) MOSFET’s notation.

The proposed voltage-to-low-current converter is shown in Figure 2a. The conversion mechanism is the same as in [18]; however, the proposed converter circuit is improved so as to support operation with low-supply voltage. Furthermore, the structure can be rail-to-rail driven.

The input signal is ∆*V_i_* and the output signal is ∆*I*. Here, saturation of all transistors is assumed. The transistor M_1_ is a DC current source that is generating the bias current *I_BIAS_*. M_2_ is a bulk-driven voltage follower. It transfers ∆*V_i_* from the bulk of M_2_ to the drain of M_1_ with a factor of about 0.25 V/V. The deviation in the drain current of M_1_ caused by the channel-length-modulation can be expressed as follows:

*I_D1_* − ∆*I_D1_* = *I_BIAS_* − ∆*I* = *I_BIAS_*(1 + *λ_p_*∆*V_SD1_*) = *I_BIAS_*(1 + *λ_p_*(∆*V_DD_* − ∆*V*_*D*1_)) (1)

where *λ_p_* is the channel-length-modulation factor in a p-channel device for given channel length. The deviation of current can also be calculated using an alternative formula.

*I_D_*_1_ − ∆*I_D_*_1_ = *I_BIAS_* − ∆*I* = *I_BIAS_*(1 + ∆*V_SD_*_1_/(*V_Ep_L*_1_)) = *I_BIAS_*(1 + (∆*V_DD_* − ∆*V_D_*_1_)/(*V_Ep_L*_1_)) (2)

where *V_Ep_* is the p-channel devices Early voltage per unit-channel-length [19], and *L*_1_ is the length of the M_1_ channel.

Assuming ∆*V_D_*_1_ ≈ 0.25∆*V_i_* and ∆*V_DD_* = 0, the I-V conversion factor, defined as transconductance *G_m_* = ∆*I*/∆*V_i_*, can be simply calculated from Equation (1) or (2):

*G_m_* = ∆*I*/∆*V_i_* ≈ 0.25·*I_BIAS_*·*λ_p_* (3)

*G_m_* = ∆*I*/∆*V_i_* ≈ 0.25·*I_BIAS_/*(*V_Ep_*·*L*_1_). (4)

When M_1_ and M_2_ are saturated, Equations (1)–(4) are valid regardless of the inversion levels in M_1_ and M_2_. However, to achieve *G_m_* of the order of 10^−9^ A/V, the inversion levels of M_1_ and M_2_ should be weak or at most moderate. The selected operating points, shown in Figure 2a, ensure the proper transistor operating range for the standard 180-nm CMOS process. For example, transistors with *L*_1_ = 200 nm, *V_Ep_* = 8.8 V/µm and *I_BIAS_* = 10 nA will give *G_m_* ≈ 1.4 nA/V (1.4 nS).

The resistance seen from the M_2_ source is much smaller than the output resistance of M_1_; thus, ∆*I* is entirely transferred from the M_1_ drain to the converter’s output (out). Next, ∆*I* is reflected in a current mirror, part of which is the diode-connected transistor M_3_.

### 2.2. Robustness to Unfavorable Factors

As long as FET remains saturated, the channel-length-modulation effect in FET is linear over a wide range of operating points [20]. Therefore, the impact of undesirable effects, such as transistor mismatch, tolerances of the manufacturing process or temperature variations on the linearity of the converter, are limited. Notwithstanding, the mentioned factors affect the *G_m_* through changes of *I_BIAS_*, *λ_p_* (or *V_Ep_L*_1_) and the follower’s gain (factor 0.25). However, measurements of the prototype converter show that the real value of *G_m_* differs from a predicted value by about only 10%.

Another aspect that requires detailed analysis involves the demonstration of the effect of temperature on *G_m_*. The increase of the current in M_1_ (∆*I*) is equal to the product of the drain voltage increase (∆*V_D_*_1_) and the drain-source conductance (*g_DS_*_1_), i.e., ∆*I* = ∆*V_D_*_1_ · *g_DS_*_1_. The conductance *g_DS_*_1_ = *I_BIAS_*/(*V_Ep_L*_1_) results from the effect of channel length modulation. Hence, *G_m_* can be expressed by the formula that is more accurate than Equation (4), i.e.,

(5) $$G_{m}=\frac{\Delta I}{\Delta V_{i}}=\frac{\Delta V_{D1}}{\Delta V_{i}}\cdot g_{DS1}=\frac{\Delta V_{D1}}{\Delta V_{i}}\cdot \frac{I_{BIAS}}{V_{Ep}L_{1}}$$

Here, *V_Ep_* and *L*_1_ do not depend on the temperature, whereas *I_BIAS_* can be stabilized. The only temperature dependent factor in Equation (5) is ∆*V_D_*_1_/∆*V_i_*, i.e., the gain of the M_2_ follower. It can be determined as

(6) $$\frac{\Delta V_{D1}}{\Delta V_{i}}=\frac{g_{mb2}}{g_{mb2}+g_{m2}+g_{DS2}+g_{DS1}+\frac{g_{DS1}g_{DS2}}{g_{m3}+g_{DS3}}}≅\frac{1}{\frac{g_{m2}}{g_{mb2}}+1}\;\approx \;0.25$$

where *g_m_*_2_ and *g_mb_*_2_ are the gate and bulk transconductances of M_2_, respectively.

Using the detailed formulas of [20], one can show that the dependence of the *g_m_*_2_/*g_mb_*_2_ ratio on temperature is relatively weak. This implies that for stable *I_BIAS_* in the M1-M2-M3 branch, *G_m_* does not depend on temperature.

## 3. Low-*G_m_* OTA (LTA)

**Figure 3:**
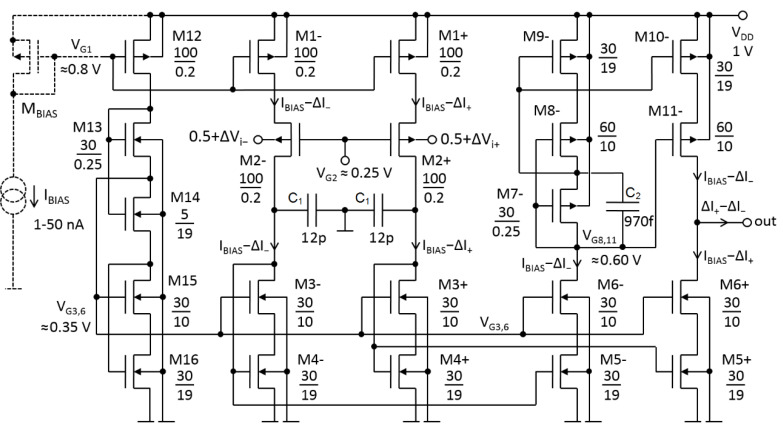
Complete electrical diagram of the low-*G_m_* OTA (LTA) prototype. The dashed lines represent an in-chip generator of V_G1_.

Based on the two converters from Figure 2a, an amplifier with differential input was developed (cf. Figure 3). The currents ΔI_+_ and ΔI_−_ from the two converters flow to the amplifier’s output (out) through two independent tracks. In other words, ΔI_+_ flows in the non-inverting path containing one mirror composed of the transistors M_3+_ to M_6+_. The ΔI_−_ flows through the inverting path containing two mirrors formed by transistors M_3−_ to M_11−_. All mirrors have a 1:1 ratio.

The amplifier’s transconductance for differential input (*G_m,diff_*) is exactly equal to *G_m_*, i.e.,

(7) $$G_{m,diff}=\frac{\Delta I_{out}}{\Delta V_{i,diff}}=\frac{\Delta I_{+}-\Delta I_{-}}{\Delta V_{i+}-\Delta V_{i-}}=\frac{G_{m}\Delta V_{i+}-G_{m}\Delta V_{-}}{\Delta V_{i+}-\Delta V_{i-}}=G_{m}.$$

The output conductance of the amplifier (*G_out_*) should be much smaller than *G_m_*, i.e., *G_out_* << 1 nS. This was achieved by using long-channel transistors and cascodes. The cascodes consisting of n-channel transistors are biased by V_G3,6_. The latter is generated by the M_12−_M_16_ branch. The “p-channel” cascodes are biased by the V_G8,11_ generated by M_7−_. All transistors in Figure 3 are standard 1.8-V thin-oxide with nominal-threshold-voltages of 0.42 V and −0.5 V for n-channel and p-channel transistors, respectively. Transistors’ dimensions (width/length expressed in µm/µm) are given in the schematic, whereas the rationale behind selecting specific dimensions is explained in Section 4.2. The schematic also shows the DC bias voltages at *V_DD_* of 1 V. Two capacitors of *C*_1_ attenuate a possible overshoot in the step transient response of the source followers M_2+_ and M_2−_ [21]. A real pole (*p*) due to the parallel combination of *C*_1_ and *g_m_*_4_ (i.e., *p* ≅ *g_m_*_4_/*C*_1_) cancels a zero (*z*) due to *C_sb_*_2_ and *g_mb_*_2_ (i.e., *z* ≅ *g_mb_*_2_/*C_sb_*_2_) in M_2+_ and M_2−._ A capacitor of *C*_2_ is a “by-pass” for an AC current flowing through the bias transistor M_7−_. It should be emphasized that these capacitors are not critical elements and have been added to the prototype circuit for research purposes only.

**Figure 4:**
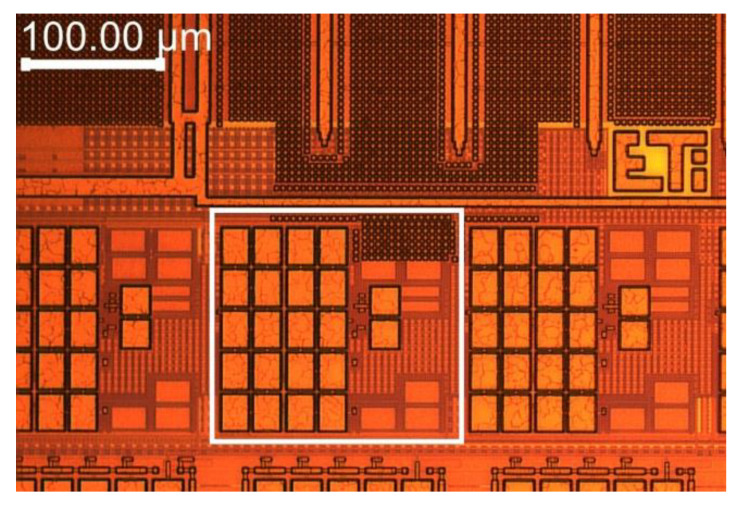
Micro photography of the prototype integrated circuit. A single piece of LTA is marked using a white rectangle.

Figure 4 shows a selected fragment of a prototype microchip that embeds LTAs. The size of a single LTA (marked using a white rectangle) is 174 µm × 156 µm. The capacitors, located close to the microstructure surface, are clearly visible. There are two 12-picofarad capacitors, each of which is composed of 10 smaller capacitors (two arrays of capacitors, each of which contains 10 components), and one 970-femtofarad capacitor (small array consisting of 2 capacitors).

## 4. Performances of the Prototype

### 4.1. Linearity of Current-Voltage Characteristics

**Figure 5:**
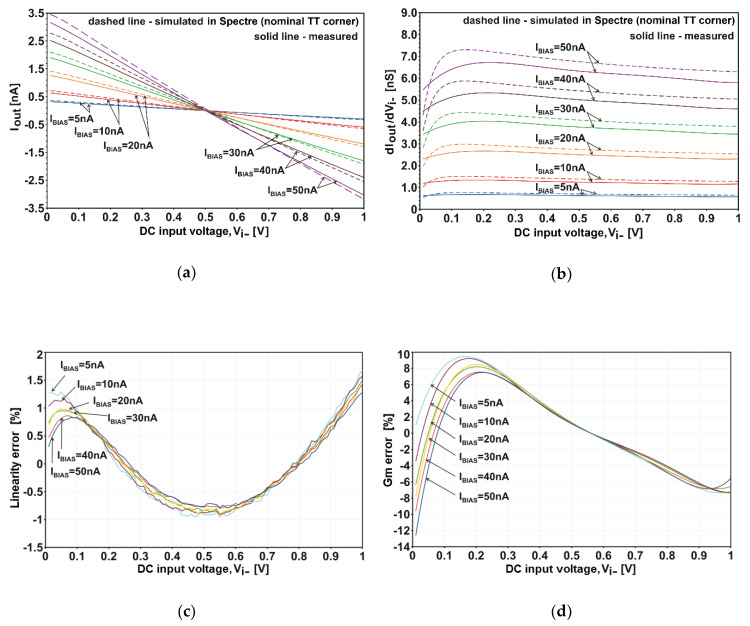
DC characteristics of the amplifier from Figure 3 at asymmetrical drive: (**a**) *I_out_* vs. *V_i−_*; (**b**) derivatives d*I_out_*/d*V_i−_*; (**c**) linearity error of the measured *I_out_* vs. *V_i−_* plots; (**d**) deviation error of the measured d*I_out_*/d*V_i−_* plots (i.e., deviation error of *G_m_*). The *V_i+_* is fixed at 0.5 V, *V_DD_* is 1 V and temperature is 27 °C.

The linearity of current-voltage characteristics was measured for unfavorable asymmetrical excitation when the input *V_i+_* was fixed at a constant potential of 0.5 V, and while the *V_i−_* was swept from 0 V to *V_DD_* = 1 V. The measured DC characteristics *I_out_* vs. *V_i−_* for several values of the *I_BIAS_* source ranging from 5 nA to 50 nA are plotted in Figure 5a. The characteristics obtained from the pre-production simulation (dashed lines) are also shown.

To calculate a linearity error of the measured *I_out_* vs. *V_i−_*, the ideal responses were first obtained using the linear regression and the least squares method. Next, the linearity error was calculated as the difference between the measured *I_out_* and the corresponding ideal *I_out_* divided by a full range of *I_out_* values, i.e., (*I_out,meas_* − *I_out,ideal_*)/*I_out,full-range_*. The calculated results (expressed in percent) were plotted in Figure 5c. The obtained error values are relatively low and range from −1% to +1.5% max. The curvature of the *I_out_* vs. the *V_i−_* plots is better visualized using derivatives d*I_out_*/d*V_i__−_*, as shown in Figure 5b.

The value of d*I_out_*/d*V_i__−_* at 0.5 V is equal to the nominal transconductance of the amplifier, i.e., *G_m_* = d*I_out_*/d*V_i__−_*|*_Vi__−_*_= 0.5_. The nominal *G_m_* can be tuned from 0.62 nA/V to 6.28 nA/V by changing the source I_BIAS_ from 5 nA to 50 nA. The percentage difference between d*I_out_*/d*V_i__−_* and the target *G_m_* (i.e., the *G_m_*-deviation error) is at most ±12% over an entire (rail-to-rail) input range. Detailed plots of the *G_m_* deviation error are in Figure 5d.

**Figure 6:**
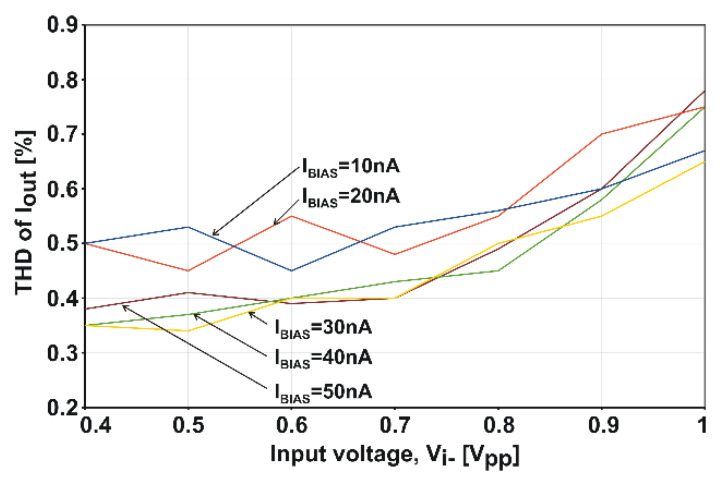
Measured THD (total harmonic distortion) of *I_out_* at an asymmetrical input driving. *V_DD_* is 1 V and *T* is 27 °C.

The amplifier was also tested for harmonic distortion. The results of measuring the harmonic content in *I_out_* for 1-kHz sinusoidal excitation are shown in Figure 6. The THD reaches 0.8% for the maximal *V_i−_* of 1 V_pp_.

**Figure 7:**
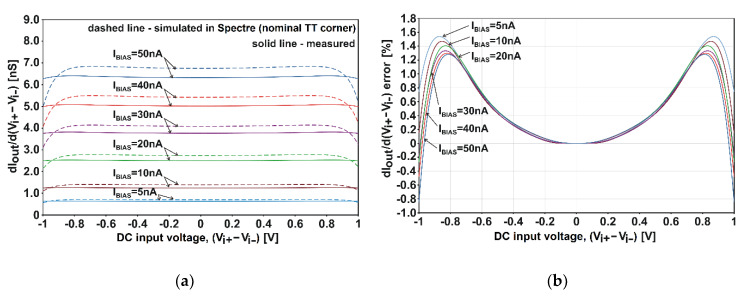
DC characteristics of the amplifier of Figure 3 at the symmetrical input drive: (**a**) derivatives d*I_out_*/d(*V_i+_* − *V_i−_*); (**b**) deviation error of the measured d*I_out_*/d(*V_i+_* − *V_i−_*) plots (i.e., deviation error of *G_m,diff_*). *V_DD_* is 1 V and *T* is 27 °C.

Clearly, nonlinearities are smaller when both inputs of the amplifier are driven symmetrically, as such a configuration ensures the highest suppression of even harmonics. It should be emphasized that in practice, an input differential signal, (*V_i+_*–*V_i−_*), is never balanced in amplifier applications with a non-differential output. However, it is worth providing results for a symmetrical excitation, because the circuit in Figure 3 can be easily equipped with a second output and adapted to “fully-balanced” applications. With symmetrical excitation with a maximum value of *V_i+_* − *V_i__−_* = 2 V_pp_, THD reaches only 0.18%. The *G_m,diff_* deviation is ±1.5% over the 2-V_pp_ input range. Detailed plots of the derivative d*I_out_*/(d*V_i+_* − d*V_i__−_*) are in Figure 7a, and the corresponding plots of the *G_m,diff_* deviation error are in Figure 7b.

**Figure A1:**
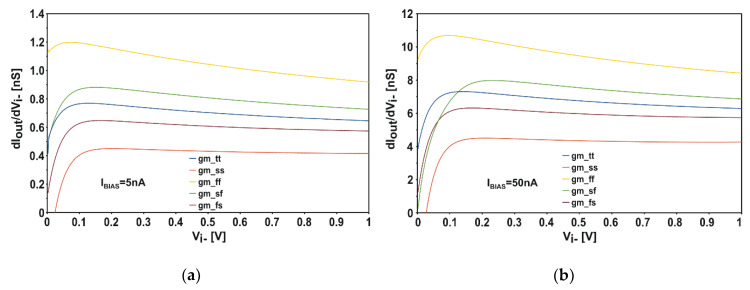
Corner simulations of the amplifier of Figure 3 at the asymmetrical input drive: (**a**) for 5-nA I_BIAS_; (**b**) for 50-nA I_BIAS_. *V_DD_* is 1 V.

**Figure A2:**
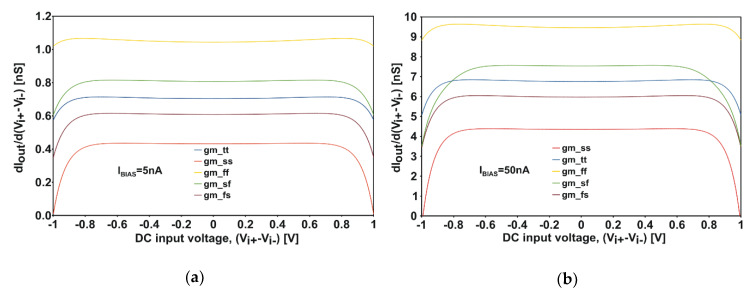
Corner simulations of the amplifier of Figure 3 at the symmetrical input drive: (**a**) for 5-nA I_BIAS_; (**b**) for 50-nA I_BIAS_. *V_DD_* is 1 V.

It should be emphasized that the prototype amplifier features better linearity than predicted by the simulations. This is clearly visible in Figure 5b in the area for *V_i−_* < 0.2 V (see also Figure A1 and Figure A2 in Appendix A). The bulk-effect (body-effect) in FET is not accurately modelled for the low potentials of bulk.

### 4.2. Frequency and Noise Characteristics

**Figure 8:**
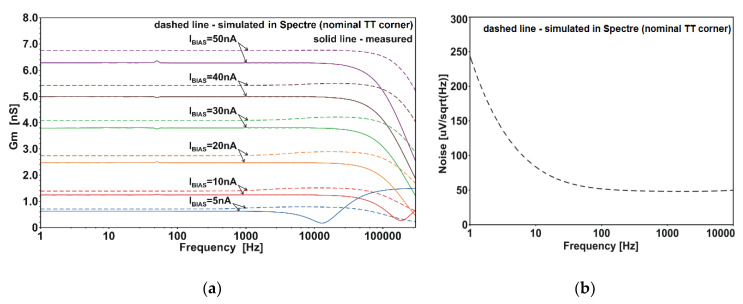
Small-signal characteristics: (**a**) of the transconductance; (**b**) of the input-referred noise. AC signal is applied to the inverting input *V_i−_*. DC levels on inputs are *V_i−_* = *V_i_*_+_ = 0.5 V. The *V_DD_* is 1 V.

A small-signal transconductance was measured in the range of 1 Hz–200 kHz. Measurements were performed separately for each of the inputs. Figure 8a shows the results only for the *V_i−_* input, because this is the worst case in terms of frequency properties (because the inverting track is longer than the non-inverting track). The values of *G_m_* for the considered frequencies are consistent with the values of d*I_out_*/d*V_i__−_* at *V_i−_* = 0.5 V obtained from the DC measurements shown in Figure 5b. The measured −3-dB frequency is lower than the one predicted in simulations by about 90–200 kHz. This is because the values of correcting capacitors used in the prototype amplifier are too large. The boost of the *G_m_* characteristic for *I_BIAS_* = 5 nA is caused by undercompensation of the measuring path.

**Figure 9:**
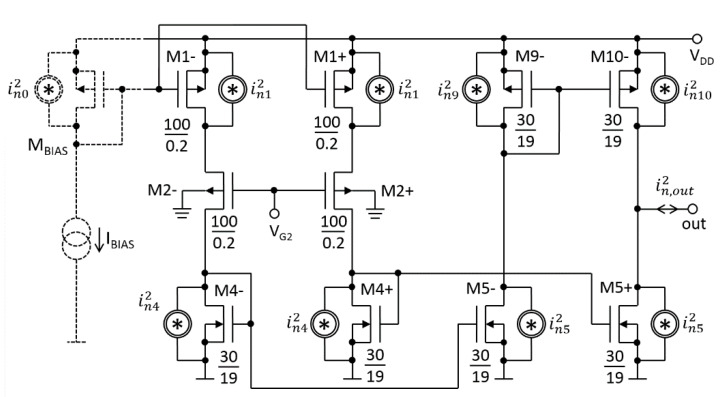
The simplified electrical diagram of the LTA for the noise calculation.

Noise characteristics obtained from the simulation are plotted in Figure 8b. In the range below 1 Hz, the 1/f noise reaches over 200 µV/(Hz)^1/2^. Above 100 Hz, the thermal noise is about 50 µV/(Hz)^1/2^. The current mirrors, particularly the transistors M_4_*_−_*, M_4+_, M_5_*_−_*, M_5+_, M_9_*_−_* and M_10_*_−_*, are the greatest contributors to the total noise. This can be explained using the electrical diagram depicted in Figure 9. The schematic contains only the transistors that significantly contribute to the total output noise. The cascode transistors (and their biasings) are removed because their contributions to the noise are minor. Also, the noise of M_2_+ and M_2_− is omitted, as those devices acts as cascodes for M_1_+ and M_1_−.

In Figure 9, the all noise currents propagate through the current mirrors with a gain of 1, so the total mean-square output noise can be expressed as the sum of the particular noise currents:

(8) $$i_{n,out}^{2}=2i_{n1}^{2}+2i_{n4}^{2}+2i_{n5}^{2}+i_{n9}^{2}+i_{n10}^{2}=2i_{n1}^{2}+4i_{n4,5}^{2}+2i_{n9,10}^{2}.$$

The noise current source (thermal + flicker) of MOSFET can be modelled as follows [22]:

(9) $$i_{n}^{2}=4kT\gamma g_{m}+\frac{K_{F}g_{m}^{2}}{fC_{OX}WL}\;[A^{2}/\mathrm{Hz}]$$

where *k* is Boltzmann’s constant, *T* is temperature, *γ* is a constant coefficient (*γ* ≈ 2/3), *K_F_* is the technological flicker-noise parameter and *f* is the frequency. Furthermore, *C_OX_* denotes gate- capacitance-per-unit-area, whereas *W* and *L* represent the width and length of MOSFET, respectively.

For weak-inversion, a MOSFET transconductance (*g_m_*) is mainly determined by a biasing current and almost does not depend on *W* and *L*. Consequently, the parameter *g_m_* is almost the same for all the devices shown in Figure 9, i.e.,

(10) $$g_{m1}≅g_{m4,5}≅g_{m9,10}=g_{m}≅I_{BIAS}q/kT$$

where *q* is the electron charge.

It should be noted that contribution of all the transistors in Figure 9 to the thermal noise—the first component of Equation (9)—is equal. Similarly, their contribution to the flicker noise—the second component of Equation (9)—is nearly equal. Notwithstanding, flicker noise can be reduced by large values of *W* and *L*.

Based on Equations (7)–(10) and Figure 2b, the input-referred noise of the LTA in Figure 3 is given as:

(11) $$e_{n,in}^{2}=\frac{i_{n,out}^{2}}{G_{m,diff}^{2}}=32qV_{Ep}^{2}L_{1}^{2}\left[\frac{16\gamma }{I_{BIAS}}+\frac{q}{k^{2}T^{2}fC_{OX}}\left(\frac{K_{FP}}{W_{1}L_{1}}+\frac{K_{FP}}{W_{9,10}L_{9,10}}+\frac{2K_{FN}}{W_{4,5}L_{4,5}}\right)\right]\;[V^{2}/\mathrm{Hz}]$$

where *K_FP_* and *K_FN_* denote the *K_F_* for the p-channel and n-channel transistors, respectively.

As can be seen from Equation (11), the low value of the total noise of LTA for the given *I_BIAS_* can be maintained using current mirrors characterized by large *W*_4,5,9,10_ and large *L*_4,5,9,10_, as well as M_1+_ and M_1−_ with large *W*_1_ and small *L*_1_. The parameter *L*_1_ was set to 0.20 µm, which is close to the technological minimum of 0.18 µm, but still sufficient to achieve a *G_m_* of the order of nS (cf. Section 2.1). It is worth noting that parameters *L*_2_ and *W*_2_ do not affect the noise. They have been set to *L*_2_ = 0.20 µm and *W*_2_ = 100 µm in order to minimize the gate-source voltage of M_2_. Similarly, parameters *L*_3,6,8,11_ and *W*_3,6,8,11_ do not affect the noise, but they have been set to over 10 µm for better matching.

### 4.3. PSRR and CMRR

**Figure 10:**
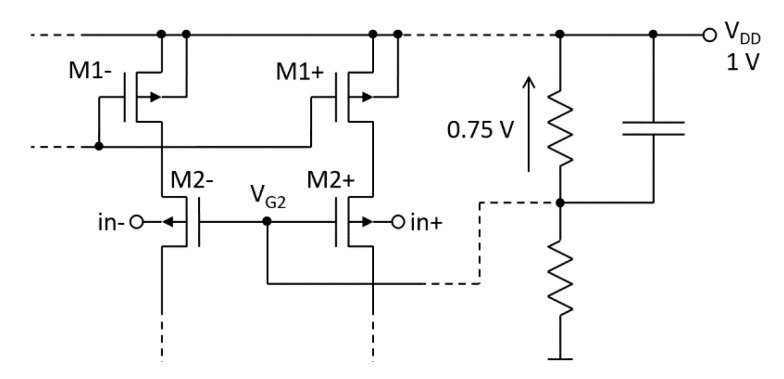
The off-chip generator of *V*_*G*2_ used for tests of the prototype LTA of Figure 3.

Owing to p-channel-based implementation, the amplifier’s input stage features small flicker noise (*K_FP_* < *K_FN_*) and supports rail-to-rail bulk driving [23]. However, for proper operation, the biasing voltage *V_G_*_2_ must be generated so that the difference *V_DD_* − *V_G_*_2_ is constant. Otherwise, the unwanted AC signal on the *V_DD_* line will be visible in ∆*I*, resulting in a reduced power-supply-rejection-ratio (PSRR). The *V_G_*_2_ can be generated in a relatively simple way, as shown in Figure 10.

The input stage does not attenuate the common component of the input differential signal. Hence, it is pseudo-differential. The common component is suppressed at the amplifier’s output node by subtracting the currents. However, mismatch of the transistors causes the inverting and non-inverting tracks to not be perfectly matched and for the currents to not be perfectly subtracted. As a consequence, the rejection of the common component is not complete. The same mechanism resulting from the mismatch also weakens the amplifier’s resistance to interferences from the *V_DD_* line. The mismatch phenomenon is random. Out of the 12 fabricated amplifier prototypes, the worst one had a CMRR (common-mode rejection ratio) of 57 dB and a PSRR of 48 dB.

### 4.4. Temperature

As already explained in Section 2.2, for stable *I_BIAS_*, the effect of temperature changes on *G_m_* is negligible. However, for the circuit of Figure 3, the temperature affects the copy of *I_BIAS_* in the non-cascoded mirrors M_BIAS_-M_1+_ and M_BIAS_-M_1−_. As indicated by the simulations, increase of the temperature from 0 °C to 70 °C, affects the increase of the *G_m_* by 15%. To put that into perspective, drift of the *G_m_* is 2.1 pS/°C and 9.7 pS/°C for *I_BIAS_* = 5 nA and *I_BIAS_* = 50 nA, respectively. The simulations were carried out under the assumption that the drift of the *I_BIAS_* source is at the level of a typical band-gap source (100 ppm/°C [24]).

### 4.5. Summary of the Performance Properties

The performance properties of the prototype are summarized in Table 1.

## 5. Application Example (Simulation Results)

**Figure 11:**
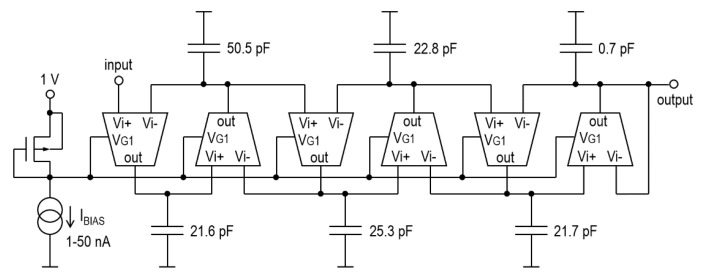
The 6th-order low-pass G_m_-C (transconductor-C) filter with Chebyshev approximation.

The LTA performance properties have been be validated using the popular application scenario, i.e., the low-pass anti-aliasing G_m_-C filter for the EEG/ECG band (0.05~100 Hz). For the system shown in Figure 1, the filter may have a smooth attenuation characteristic, i.e., its order may be low (from 2 to 4) [25,26,27,28]. Furthermore, approximation can be realized using Butterworth [28,29] or Bessel functions. Advanced, “sharp” filtration is performed in a digital signal processor (DSP). The advantage of smooth filters is that they do not have significant requirements with respect to the performance of transconductance amplifiers. On the other hand, such filters are often insufficient for performing thorough tests of amplifier circuits. Therefore, in this example, a more demanding 6th order Chebyshev filter is used (cf. Figure 11).

**Figure 12:**
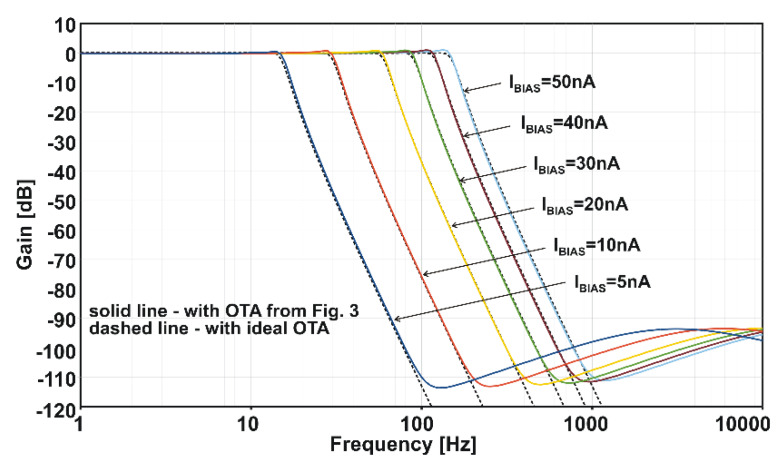
Simulated amplitude responses of the filter from Figure 8.

Under the assumption that transconductors are ideal, the frequency parameters of the filter in Figure 11 are as follows: the gain in the pass-band is 1 *v/v* (0 dB), the amplitude slope is −120 dB/dec and the attenuation in the stop-band is infinite. In reality, however, non-ideal parameters of transconductors that include finite output resistance, as well as parasitic zeros and transmittance poles, contribute to a more gradual slope of the characteristic and limited stop-band attenuation. Figure 12 shows a comparison of an ideal filter (dashed lines) with the one that implements the transconductors shown in Figure 3 (solid lines). The corner frequency of the filter can be tuned from 14.6 Hz to 144.8 Hz by changing the *I_BIAS_* between 5 nA and 50 nA. As can be seen, the considered transconductors accurately reproduce characteristics of the filter down to −90 dB, which is a very good result when it comes to analog G_m_-C filters. When it comes to the large-signal properties, the output THD is below 1% (−40 dB) for sinusoidal excitation with a V_pp_ amplitude of 0.6 and frequency ten-fold lower than the corner one. When the excitation frequency is close to the corner one, i.e., near the end of the pass-band, the output distortions HD_2_ = −36 dB and HD_3_ = −55 dB, respectively.

## 6. Discussion and Conclusions

Linear LTAs are realized using various techniques such as current division (current splitting), current cancellation, bulk driving, source degeneration or floating gates. The examples can be found in many literature references, e.g., [2,3,8,9,10,12,13,14,15,16,17,30,31]. To the best of authors’ knowledge, to date, the channel-length-modulation effect for LTA realization has been reported only in [18]. The prototype amplifier solution in [18] is interesting, but it operates with a relatively high supply voltage of 5 V.

The proposed solution has been compared against state-of-the-art circuits from the literature comprising the linear transconductors with possibly close *G_m_* values. The results are collected in Table 2. Most of the solutions are characterized by differential input/output and are only symmetrically driven. In such conditions, the proposed transconductor is the only one that maintains linearity when driven by a signal (2 V_pp_) greater than the supply voltage (1 V). However, in the proposed solution, the noise is relatively high and, despite maintaining linearity in a wide range, it does not feature improved SNR as compared other solutions. On the other hand, the proposed circuit features improved performance in terms of low power consumption (0.3 µW) and low supply voltage (1 V). Temperature parameters cannot be compared because this parameter is not reported in majority of the available literature.

The presented transconductor solution is dedicated to working in a system where high gain is provided by the input LNA. From this perspective, the noise of an antialiasing transconductor-C filter is not of primary concern. Nevertheless, the noise in the proposed transconductor can be reduced through implementation in a single stage topology. It should be worth noting, however, that the single-stage topology is characterized by a narrower driving range. Therefore, choice between the single- and multi-stage topologies is a compromise between maintaining a low-noise and high-driving amplitude. The use of low-noise analog-dedicated CMOS technology (with lower technological parameters *K_FN_* and *K_FP_* in Equation (11) can also be considered to address the mentioned challenges.

## Figures and Tables

**Table 1:** Parameters of the prototype LTA at *V_DD_* = 1 V and 27 °C.

Parameter	Simulated	Measured
Technology/Vendor	Standard 180 nm CMOS 1P6M/TSMC	
Physical dimensions ^1^	174 µm × 156 µm	
Supply voltage V_DD_	1 V (min. 0.8 V, max. 1.8 V)	
Average current consumption ^1^	32–290 nA	28–270 nA
G_m_ tuning range (I_BIAS_ range 5–50 nA)	0.7–6.75 nS	0.62–6.28 nS
G_m_ temperature drift ^2^	2.1 pS/°C @ I_BIAS_ = 5 nA 9.7 pS/°C @ I_BIAS_ = 50 nA	- -
Input common-mode range	0.1–1 V	0–1 V (rail-to-rail)
THD of I_out_ non-symmetrical driving symmetrical driving	2.4% @ 1 V_pp_, 1% @ 0.64 V_pp_ 0.47% @ 2.0 V_pp_	0.8% @ 1 V_pp_ 0.18% @ 2 V_pp_
Gm deviation (linearity) error non-symmetrical driving symmetrical driving	±22% @ 1 V_pp_ ±12% @ 2 V_pp_	±12% @ 1 V_pp_ ±1.5% @ 2 V_pp_
Input-reffered noise	760 µV_RMS_ (integrated over 1–100 Hz)	-
Signal to noise ratio (SNR) non-symmetrical driving symmetrical driving	49.5 dB @ THD = 1% 59.3 dB @ THD = 0.47%	- -
CMRR, PSRR	min. 56 dB, 47 dB ^3^	min. 57 dB, 48 dB ^4^
Input offset voltage (V_OS_)	max. ± 25 mV ^3^	25–50 mV ^4^
Mismatch-induced deviation of Gm	max. ± 4.5% ^3^	-

^1^ Without the circuits drawn with dashed lines in Figure 3. ^2^ G_m_ deviation is 15% max when temperature varies from 0 to 70 °C. ^3^ 200 Monte Carlo runs. ^4^ For 12 fabricated amplifier samples.

**Table 2:** Comparison of linear LTAs (linear low-G_m_ OTAs).

Parameter	This Work	[12] (BD+CD Case)	[16] (Simulated)	[17]	[18]
Type of input/output	diff./single	diff./single	diff./diff.	diff./single	diff./diff.
G_m_	0.62–6.28 nS	9.4 nS	39.5–367.2 nS	0.46–82 nS	30 pS–25 µS
Supply voltage	1 V	2.7 V (±1.35 V)	5 V (±2.5 V)	1.5 V	5 V (±2.5 V)
Power consumption	<0.3 µW (28–270 nW)	4.05 µW (sim.)	160 µW	<1 µW	<300 µW
Input comm.-mode range	rail-to-rail	-	-	rail-to-rail	-
Linear range for symmetrical input	2 V_pp_ @ 0.18% THD	0.9 V_pp_ @ 1% HD_3_	2 V_pp_ @ 0.13% THD	1.2 V_pp_ @ 1% THD	2.6 V_pp_ @ 1% THD
Input-referred noise	760 µV_RMS_ (sim.) (1–100 Hz)	104.7 µV_RMS_ (0.01–10 Hz)	332 µV_RMS_ (10–30 kHz)	110 µV_RMS_ (1–100 Hz)	635 µV_RMS_ (1 Hz–2 MHz)
SNR	59.3 dB (sim.) @ 0.47% THD	69.6 dB @ 1% HD_3_	~66.5 dB @ 0.13% THD	70 dB @ 1% THD	62 dB @ 1% THD
CMRR/PSRR	56 dB/47 dB	-	>44.8 dB/n.a.	-	>80 dB/>80 dB
CMOS process	0.18 µm	1.2 µm	0.35 µm	0.8 µm	0.35 µm
Layout area	0.027 mm^2^	0.22 mm^2^	0.006 mm^2^	0.04 mm^2^	0.046 mm^2^

## Appendix A

Figure A1 and Figure A2 present simulation results obtained for all corners (worst case analysis). The responses represent supplementary data to the characteristics of Figure 5b and Figure 7b.
